# Single-cell RNA sequencing revealed cell heterogeneity in sagittal suture mesenchyme

**DOI:** 10.3389/fcell.2026.1725375

**Published:** 2026-01-20

**Authors:** Chengyan Ren, Kai Sun, Ran Wu, Chenxin Geng, Jiangping Chen, Hu Zhao, Weihui Chen

**Affiliations:** 1 Department of Oral and Maxillofacial Surgery, Fujian Medical University Union Hospital, Fuzhou, China; 2 State Key Laboratory of Oral & Maxillofacial Reconstruction and Regeneration, Key Laboratory of Oral Biomedicine Ministry of Education, Hubei Key Laboratory of Stomatology, School & Hospital of Stomatology, Wuhan University, Wuhan, China; 3 Chinese Institute for Brain Research, Beijing, China; 4 Department of Prosthodontics, Peking University School and Hospital of Stomatology, National Center for Stomatology, National Clinical Research Center for Oral Diseases, National Engineering Research Center of Oral Biomaterials and Digital Medical Devices, Beijing, China; 5 Department of Stomatology, Oromaxillofacial Head and Neck Surgery, Huashan Hospital, Fudan University, Shanghai, China

**Keywords:** cell heterogeneity, sagittal suture, single-cell RNA sequencing, suture mesenchymal stem/stromal cells, transient amplifying cells

## Abstract

**Introduction:**

The formation and homeostatic maintenance of cranial sutures rely on cellular activities within the suture mesenchyme. However, how mesenchymal stem/stromal cells (MSCs) rapidly and extensively contribute to suture and cranial development remains insufficiently explored.

**Methods:**

We integrated 10x Genomics and Smart‐seq3 single‐cell transcriptomic sequencing to analyze cellular subpopulations in the sagittal suture mesenchyme. CytoTRACE2 analysis was performed to quantitatively assess the differentiation status of each cell population. We further characterized the progenitor with characteristics of transient amplifying cells (TACs) via 5‐ethynyl-2’‐deoxyuridine (EdU) assays, in situ hybridization, and lineage tracing using *Ki67Cre^ERT2^;tdTomato* mice. Through bioinformatics analysis based on sequencing data, we filtered transcription factors of key cell populations.

**Results:**

Smart‐seq3 showed higher sequencing depth and improved capture efficiency for target cell populations. Then, we identified a proliferative progenitor population in the sagittal suture that exhibited features of TACs. These TACs were a committed, proliferative direct lineage of suture mesenchymal stem/stromal cells (SuSCs) and responsible for rapid development of cranial structures. Additionally, *Erg* and *E2f7/8* were expressed in SuSCs and TACs, respectively. Among these, *Erg* downstream targets participated in biological processes governing MSCs and bone morphogenesis processes, while *E2f7/8* downstream targets primarily regulate the cell cycle.

**Discussion:**

This study provides the first identification of TACs within the developing cranial suture niche and elucidates key regulatory genes and signaling networks in SuSCs and TACs, thereby providing a theoretical framework for understanding the mechanisms underlying cranial suture formation and homeostasis.

## 1 Introduction

Cranial sutures function as dynamic hubs where stem cell biology, bone formation, and mechanical forces interact, guiding skull morphogenesis across the lifespan (Roth et al., 2022). They comprise two bone ends separated by intervening fibrous tissue which derives from embryonic mesenchyme (Mishina and Snider, 2014). Suture mesenchymal stem/stromal cells (SuSCs) are a subgroup of mesenchymal stem/stromal cells (MSCs) located within the cranial sutures (Li et al., 2021). SuSCs possess self-renewal and multi-lineage differentiation potential, including osteogenic and chondrogenic differentiation (Zhao et al., 2015; Maruyama et al., 2016). They serve as a critical cellular source during cranial suture and surrounding tissue formation, and are essential for maintaining the homeostasis of the suture microenvironment (Maruyama et al., 2016; Farmer et al., 2021; Holmes et al., 2021). Therefore, exploring and understanding the regulatory mechanisms related to the differentiation process of SuSCs is essential.

Transit-amplifying cells (TACs) are a population of cells that have rapid proliferation but low self-renewal capacity (Zhao et al., 2014). Their differentiation level is between that of MSCs and mature differentiated cells, and they are spatially located adjacent to upstream MSCs (Clayton et al., 2007; Zhao et al., 2014; Walker et al., 2019). As the direct progeny of MSCs that amplify in number before differentiating into specialized cells, TACs can rapidly amplify and then differentiate, providing sufficient cells for organ development, regeneration, and repair process (An et al., 2018; Jing et al., 2021; Cancedda and Mastrogiacomo, 2023). Previous studies have found a group of quiescent MSCs in the root apex region of mouse incisors, which first transformed into TACs and rapidly proliferated, followed by further differentiation into functional cells that could form mineralized tissue and maintain continuous growth of the incisor throughout its life (Li et al., 2015; Zhao et al., 2018). However, whether a cell population similar to TACs exists in the cranial sutures to meet the cellular demand for cranial suture formation has not yet been studied.

In recent years, the widespread application of transcriptome sequencing technology at single-cell resolution has provided a new perspective for elucidating the developmental mechanisms of cranial sutures (Li et al., 2023a). However, due to the narrow distance between the bone ends on both sides, the total amount of cells in cranial sutures is relatively low, which poses significant challenges to the cell capture efficiency and sequencing depth of single-cell sequencing (Li et al., 2023b). 10x Genomics is currently the most widely used sequencing method for cranial suture-related research at single-cell resolution. It can efficiently process comprehensive cell samples, achieving high-throughput cell capture and detection (Ziegenhain et al., 2017; Ashton et al., 2021). Smart-seq is a microplate-based single-cell sequencing method that achieves full-length cDNA synthesis through a sequence conversion mechanism at the 5′end of the RNA template. The recently proposed Smart-seq3 technology by Hagemann-Jensen et al. also introduces a unique molecular identifier (UMI) counting strategy at the 5′end, significantly enhancing sequencing efficiency and providing new technical support for the fine classification of cell types and subpopulations (Hagemann-Jensen et al., 2020).

In this study, we combined two single-cell transcriptome sequencing technologies, including 10x Genomics and Smart-seq3, to analyze the cell subpopulations in the cranial suture mesenchyme. We characterized the gene expression of SuSCs, conducted a preliminary identification of TACs in the cranial sutures, and performed an initial analysis of the signaling regulatory networks in SuSCs and TACs based on single-cell transcriptome sequencing data.

## 2 Materials and methods

### 2.1 Animal

*Gli1-Cre* ^*ERT2*^ (JAX#007913), *Ki67-Cre* ^*ERT2*^ (JAX#029803) and *tdTomato* (JAX#007914) mouse lines were used in this study. The mice were housed under specific pathogen-free conditions. Genotyping was conducted using the One Step Mouse Genotyping Kit (Vazyme, Nanjing, China) on tail samples. Primer sequences were listed in Supplementary Table S1. All animal studies were approved by Chinese Institute for Brain Research (LARC‐T020).

### 2.2 Slice preparation

Skulls of postnatal mice were dissected and fixed in 4% paraformaldehyde (PFA) overnight at 4 °C. Subsequently, the samples were decalcified with 10% EDTA depending on mouse age in days. For paraffin sections, decalcified samples were dehydrated and embedded in paraffin. The blocks were sectioned (8 μm) using a microtome and mounted on slides (CITOTEST, Nanjing, China) for H&E staining which was performed according to the standard protocol. For frozen sections, samples were embedded in optimal cutting temperature compound (OCT) for support during sectioning.Thin sections (15 µm) were cut using a cryostat maintained at −20 °C. The resulting frozen sections were mounted onto glass slides for subsequent analysis.

### 2.3 10x single cell RNA-seq

**FIGURE 1:**
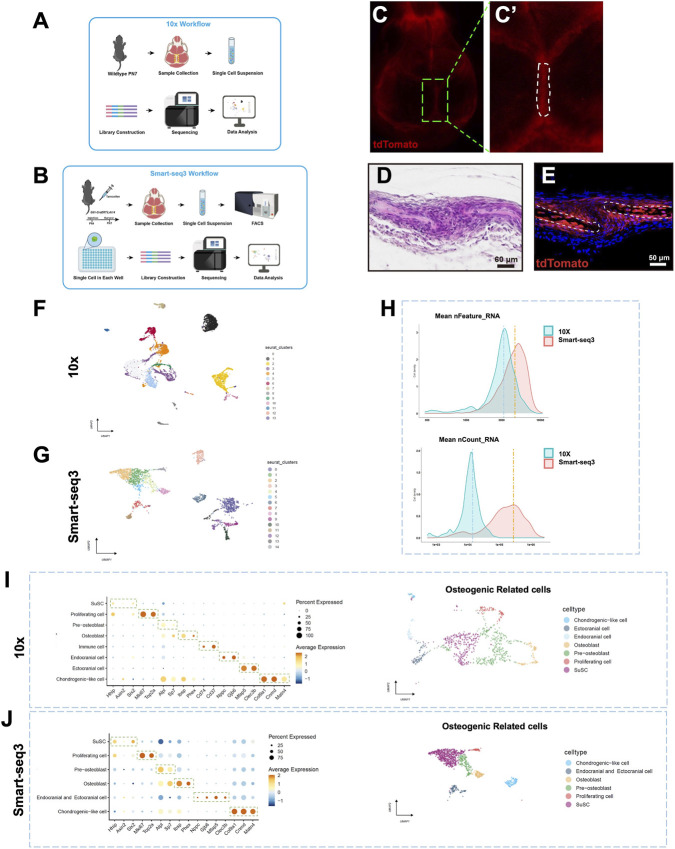
Characterization of Osteogenic-Related Cell Heterogeneity in the Sagittal Suture. **(A,B)** Summary of the sample collection and analysis workflow. **(C,C’)** Expression of tdTomato signal in the skull of *Gli-Cre* ^*ERT2*^ *;tdTomato* mice at 3 days after tamoxifen induction. **(D)** HE staining of the sagittal suture in PN7 wild-type mice. **(E)** Expression of tdTomato signal within the sagittal suture of *Gli-Cre* ^*ERT2*^ *;tdTomato* mice at 3 days after tamoxifen induction. **(F,G)** The UMAP visualization of cells for 10x group and Smart-seq3 group, respectively. **(H)** The comparison between 10x group and Smart-seq3 group for mean nFeature RNA and mean nCount RNA. **(I,J)** The characteristic expression genes of each subpopulation and UMAP visualization of osteogenic related cells for 10x group and Smart-seq3 group, respectively.

The sagittal suture was dissected from C57BL/6J mice (see Figure 1A). The library consisted of the sagittal sutures from four PN7 mouse pups. The digestion of pooled suture samples and the preparation of cell suspensions were conducted consistent with the aforementioned steps. The cell viability was assessed using acridine orange/propidium iodide (AO/PI, ApexBio, USA) staining on the Luna FL (Aligned Genetics, South Korea). Single-cell libraries were prepared using the BD Rhapsody Single-Cell Analysis System (BD, USA), according to the manufacturer’s protocol. and cDNA quality was evaluated by fragment analysis (Qsep100; Bioptic). RNA sequencing was carried out using the Illumina NovaSeq X Plus System. The 10x scRNA sequencing was performed by Glbizzia Biosciences (China).

### 2.4 Smart-seq3

Tamoxifen (Sigma-Aldrich, USA) was suspended in corn oil (Sigma-Aldrich) at 20 mg/mL and injected intraperitoneally to *Gli1-Cre* ^*ERT2*^; *tdTomato* mice at a dosage of 75 μg per gram of body weight daily at PN4. The sagittal suture was dissected from PN7 *Gli1-Cre* ^*ERT2*^; *tdTomato* mice (see Figure 1B). Samples were digested in a-MEM at 37 °C with 0.2% type II collagenase (Sigma-Aldrich), 0.2% type II dispase (Sigma-Aldrich), and 1 U/μL deoxyribonuclease I (Worthington, USA) in DMEM/F12 (Gibco, USA). The digestion was conducted in a water bath for 45 min, with thorough shaking every 15 min. Then, the suspensions were placed on ice and supplemented with 2% FBS to terminate the digestion. The cell suspensions were filtered through a 70 µm filter (Novbio, USA), centrifuged at 400 *g* for 5 min, washed with PBS/1% BSA, and then resuspended in PBS/1% BSA. Red blood cell lysis was performed following the centrifugation of the filtered cells (Solarbio, China). Then, target cells were collected through fluorescence-activated cell sorting (FACS). Calcein Violet 450 AM (Invitrogen, USA; 0.005 mM) was used to mark live cells. Then, tdTomato-positive live cells were sorted to each well containing lysis reaction mix in 384 well plate. Library preparation and sequencing were performed according to previously reported workflow in Genomics Center of Chinese Institute for Brain Research (Hagemann-Jensen et al., 2022).

### 2.5 Analysis of single cell sequencing

The downstream steps were performed using Seurat in RStudio (version 4.4.1). All cells were filtered to retain those with at least 200 detected genes (nFeature RNA) and less than 10% mitochondrial transcripts. Uniform Manifold Approximation and Projection (UMAP) was employed for dimensionality reduction using Seurat. CytoTRACE2 was applied to identify differentiation states across all cell types. SCENIC analyses were used to identify key and unique transcription factors in the populations of SuSCs and proliferating cells. Gene ontology (GO) enrichment analysis was performed using clusterProfiler with the org.Mm.e.g.,.db database. Genes lacking GO Biological Process (BP) annotations or with unresolvable identifiers were excluded. Cell cycling scoring was applied to quantify the proliferative status of individual cell population.

### 2.6 Immunofluorescence assays

Rinse the frozen sections in 1x phosphate buffered saline (PBS), for OCT removal. Then, sections were blocked for 1 h at room temperature in blocking solution (ZSGB-BIO, China), and incubated with primary antibodies against Sp7 (HuaBio, China) or Ki67 (Abcam, UK) overnight at 4 °C. After washing three times with PBS, sections were then incubated with secondary antibodies (ZSGB-BIO) for 1 h at room temperature. DAPI (ZSGB-BIO) was used to stain cell nucleus. Images were taken (40x), using a confocal microscope (Leica SP8; Germany).

### 2.7 EdU proliferation assays

Mice were sacrificed at 2, 4, 8 and 12 h after intraperitoneal 5-Ethynyl-2′-deoxyuridine (EdU) injection (10 mg/kg; RiboBio, China). Anti-Sp7 or anti-Ki67 staining was performed as described above (Immunofluorescence assays). After incubated with secondary antibody, the EdU labeling was performed using the using Click-iT Apollo 567 Stain Kit (RiboBio), according to the manufacturer’s instructions. Sequential imaging was performed on a Leica TCS SP8 LAS X microscope (40×/1.30 NA HCX PL APO CS2 oil-immersion objective). EdU and tdTomato were excited individually with 488 nm and 540 nm laser lines at 1.5% transmission. Their emission was captured through spectrally distinct detection paths: EdU signal was collected at 493–540 nm with a PMT (Gain 650), and tdTomato fluorescence was routed to a HyD detector set to 555–610 nm (Gain 50). A sequential scanning strategy was employed to eliminate spectral cross-talk.

### 2.8 *In situ* mRNA hybridization

Secreted Frizzled-Related Protein 2 (*Sfrp2*), microfibrillar-associated proteins (*Mfap4*) and marker of proliferation Ki-67 (*Mki67*) mRNA localization was assessed using the PinpoRNA multiplex Fluorescent RNA *in-situ* hybridization kit (GD Pinpoease Biotech Co. Ltd., China). Following standard deparaffinization and rehydration of paraffin sections, endogenous peroxidase activity was blocked with Pre-A solution at room temperature. Target RNA accessibility was enhanced through protease treatment prior to hybridization with specific probes for 2 h at 40 °C. Signal amplification proceeded through three sequential reaction steps as per the manufacturer’s protocol. Target RNA molecules were subsequently labeled with green fluorescence using a tyramine-based fluorescent substrate. Fluorescent signals were visualized and captured using a Leica SP8 confocal microscope.

### 2.9 Statistics analysis

Statistical significance was assigned for *P* ≤ 0.05. Statistical analysis was performed using a one-way analysis of variance (ANOVA). The number of cells was calculated by ImageJ. All statistical calculations were performed using the Prism 10 (GraphPad) software package.

## 3 Results

### 3.1 Smart-seq3 shows higher sequencing depth and improved capture efficiency for target cell populations

The workflow of 10x and Smart-seq3 sequencing is illustrated (Figures 1A,B). The dissection range of sagittal suture for Smart-seq3 sequencing was determined by observing tdTomato-positive area in gross specimens (Figures 1C,C’). On tissue sections, tdTomato-positive cells at PN7 were predominantly distributed in the suture, parietal bone, and partial endocranial and ectocranial cells (Figures 1D,E). After processing the sequencing data with the Seurat package, preliminary clustering results for all cells were obtained (Figures 1F,G). The mean nFeature RNA and mean nCount RNA represent the average number of gene types and the average gene count captured per cell, respectively, serving as key indicators of sequencing depth. The results showed that the mean nFeature RNA was 3,224 for the 10x group and 4,553 for Smart-seq3 group, while the mean nCount RNA values were 14,160 and 276,431 for the 10x and Smart-seq3 groups, respectively (Figure 1H).

Based on known marker genes, we identified and excluded non-osteogenic related cell populations, including immune cells, endothelial cells, and pericytes (Supplementary Figures S1, S2), and subsequent analyses focused primarily on the osteogenic-related cells. We noted that *Cd37*/*Cd74*-positive immune cells still remained in the 10x group after initial filtration (Figure 1I), hence this population was further excluded. Based on the characteristic expression genes of each subpopulation, we annotated all the celltypes of osteogenic-related cells as Pre-osteoblast, Osteoblast, Chondrogenic-like cell, Endocranial cell, and Ectocranial cell. Additionally, based on the high expression of *Hhip*, *Six2*, and *Axin2* (Farmer et al., 2021; Holmes et al., 2021), we defined SuSC (Figures 1I,J). We identified Proliferating cell population by integrating cell cycling scoring with the characteristic expression patterns of the Mki67 and Top2a genes, which exhibited highest score of G2/M and S phase (Figures 1I,J; Supplementary Figure S3).

We then analyzed the number of osteogenic-related cells obtained from the two sequencing methods. The Smart-seq3 group detected a total of 1,115 osteogenic-related cells, accounting for 49.5% of all tdTomato-positive cells captured. In contrast, the 10x group detected only 984 osteogenic-related cells, representing just 14.5% of the total captured cells (Table 1). Additionally, the Smart-seq3 group captured 608 SuSCs, with a proportion of 27.0%, which was higher than that of the 10x group (303, 4.5%) (Table 2).

**TABLE 1:** The number of cells retained through primary filtration.

Cell type	10x	Smart-seq3
Osteogenic related cells	984 (14.5%)	1,115 (49.5%)
Non-osteogenic cells	5,823 (85.5%)	1,137 (50.5%)
Total	6,807	2,252

**TABLE 2:** The cell number of each subcluster in osteogenic-related cells.

Cell type	10x	Smart-seq3
SuSCs	303 (4.5%)	608 (27.0%)
Endocranial and ectocranial cells	143 (2.1%)	97 (4.3%)
Pre-osteoblasts	340 (5.0%)	155 (6.9%)
Osteoblasts	89 (1.3%)	97 (4.3%)
Proliferating cells	84 (1.2%)	45 (2.0%)
Chondrogenic-like cells	25 (0.4%)	113 (5.0%)

### 3.2 Single-cell transcriptome sequencing reveals the differentiation status of subcellular groups within the sagittal suture

**FIGURE 2:**
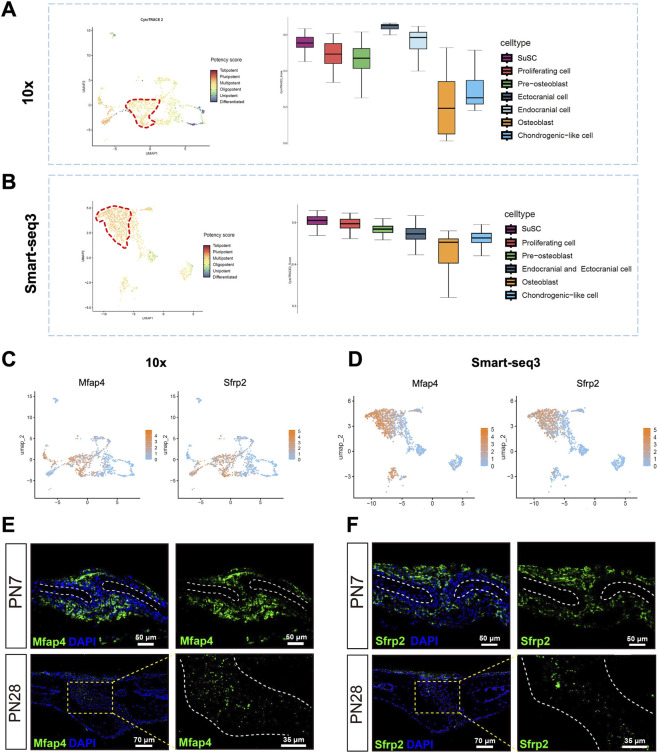
The CytoTRACE2 analysis result and the *in situ* hybridization of *Sfrp2* and *Mfap4* transcript in sagittal suture. **(A,B)** The CytoTRACE2 analysis of osteogenic related cells for 10x group and Smart-seq3 group, respectively. **(C)** The UMAP visualization of *Sfrp2* and *Mfap4* in osteogenic related cells based on 10x data. **(D)** The UMAP visualization of *Sfrp2* and *Mfap4* in osteogenic related cells based on Smart-seq3 data. **(E)** The *in situ* hybridization of *Mfap4* transcript in sagittal suture of PN7 and PN28 wild type mice. **(F)** The *in situ* hybridization of *Sfrp2* transcript in sagittal suture of PN7 and PN28 wild type mice.

Using CytoTRACE2 analysis to assess the differentiation status of each cell population, we observed a gradual increase in differentiation levels from SuSCs and Proliferating cells to Pre-osteoblasts and Osteoblasts in both the 10x and Smart-seq3 groups (Figures 2A,B). Chondrogenic-like cells exhibited slightly lower differentiation levels compared to Osteoblasts. Notably, in the 10x group, Ectocranial and Endocranial cells displayed the lowest differentiation state among all cell populations (Figure 2A). In contrast, in the Smart-seq3 group, Ectocranial and Endocranial cells showed higher differentiation levels than SuSCs and Proliferating cells, positioning them between Pre-osteoblasts and Osteoblasts (Figure 2B).

### 3.3 *Sfrp2* and *Mfap4* transcripts were highly in SuSCs

To verify that the sequencing data matched the spatial distribution, we analyzed the top five highly expressed genes in SuSCs (Supplementary Figures S4, S5). UMAP plots showed that only *Sfrp2* and *Mfap4* were highly enriched in SuSCs with no significant expression in Pre-osteoblasts, Osteoblasts and Proliferating cells, while both genes were detected in Ectocranial and Endocranial cells individually (Figures 2C,D; Supplementary Figure S6). Furtherly, we validated the spatial expression patterns of *Sfrp2* and *Mfap4* transcripts in the sagittal suture at PN7 using *in situ* hybridization. The results revealed that *Mfap4* was mainly expressed in the center of sagittal suture and in the endocranial region, with low-level expression in ectocranial cells (Figure 2E); *Sfrp2* was expressed in the central region of sagittal suture and distributed in the ectocranial area (Figure 2F), which is consistent with the sequencing annotation results. We further examined the expression patterns of *Mfap4* and *Sfrp2* in the sagittal suture at PN28 and found that, compared with the active growth stage at PN7, the transcript expression levels of both genes were markedly reduced at PN28, while maintaining certain expression in the central area of the sagittal suture (Figures 2E,F). In addition, at this stage, *Mfap4* and *Sfrp2* were also expressed in ectocranial and endocranial cells around the suture at a relative low level (Figures 2E,F).

### 3.4 TACs exist in the sagittal suture

**FIGURE 3:**
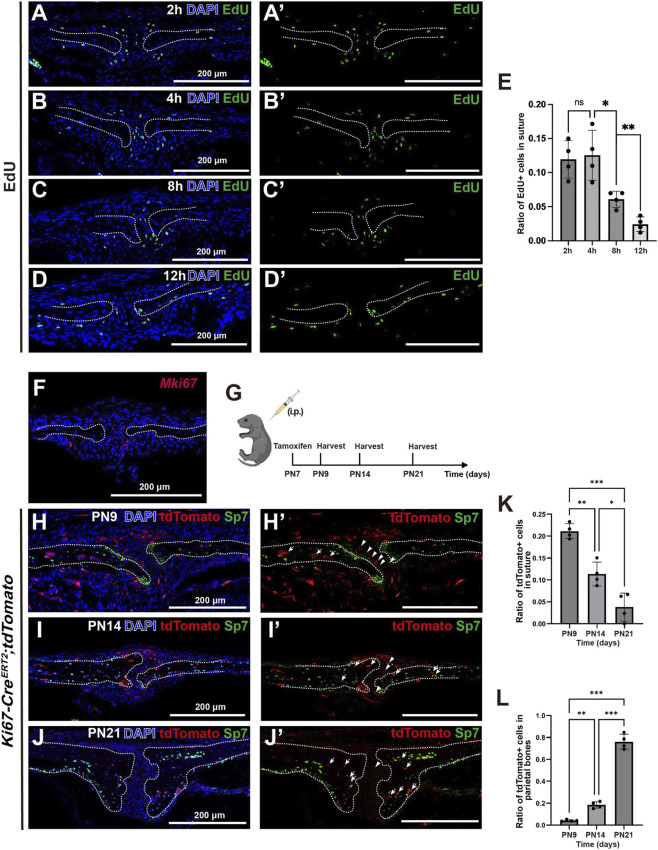
The spatial distribution of proliferating cell populations within sagittal suture. **(A–D)** The results of EdU assay within sagittal suture of PN7 wild type mice at 2, 4, 8, 12 h after intraperitoneal injection of EdU. **(A′–D′)** The signals of EdU within sagittal suture in **(A–D)**. **(E)** Quantitative analysis of the ratio of EdU-positive cells in mesenchyme of sagittal suture. **(F)** *In situ* hybridization of *Mki67* transcript in sagittal suture of PN7 wild type mice. **(G)**The scheme of intraperitoneal injection of tamoxifen and sample harvest time point. **(H,H′)** The localization of tdTomato- and Sp7-positive cells in within sagittal suture of PN9 *Ki67-Cre* ^*ERT2*^ *;tdTomato* mice. **(I,I′)** The localization of tdTomato- and Sp7-positive cells in within sagittal suture of PN14 *Ki67-Cre* ^*ERT2*^ *;tdTomato* mice. **(J,J′)** The localization of tdTomato- and Sp7-positive cells in within sagittal suture of PN21 *Ki67-Cre* ^*ERT2*^ *;tdTomato* mice. Arrowheads indicate tdTomato-positive cells within sagittal suture. Arrows indicate tdTomato-positive cells within parietal bones. **(K)** Quantitative analysis of the ration of tdTomato-positive cells in sagittal suture mesenchyme. **(L)** Quantitative analysis of the ration of tdTomato-positive cells in parietal bones. Scale bar = 200 μm. n = 4 per group. Student’s t-test was used. *P < 0.05; **P < 0.01; ***P < 0.001.

The EdU assay is widely used for assessing cell proliferative activity (Contreras et al., 2025). We first employed EdU assay to detect the distribution pattern of proliferating cells within the sagittal suture. At 2 h and 4 h post-administration, EdU-positive cells in the sagittal suture were distributed between the central region of suture mesenchyme and osteogenic fronts, accounting for approximately 10%–15% of the total cell population (Figures 3A,B,A′,B′,E). By 8 h and 12 h post-administration, this proportion decreased significantly to a low level (Figures 3C,D,C′,D′,E). At 12 h post-administration, the results of EdU and Sp7 co-labeling demonstrated the presence of EdU and Sp7 double-positive cells in the parietal bone (Supplementary Figure S7), indicating that EdU-positive cells were capable of undergoing osteogenic differentiation. Furthermore, we examined the co-localization of EdU and Ki67 in the sagittal suture and found that at 2 h and 4 h post-administration, approximately 60% of the EdU-positive cells in the sagittal suture co-expressed Ki67 (Supplementary Figure S8). On the other hand, *in situ* hybridization detection of cells expressing *Mki67* transcripts revealed that *Mki67*-positive signals were also primarily localized lateral region of sagittal suture (Figure 3F). Furthermore, to investigate the long term contribution of these cells, we performed lineage tracing of proliferating cells in the sagittal suture using *Ki67-Cre* ^*ERT2*^ *;tdTomato* mice (Figure 3G). We found that at PN9, tdTomato-positive cells were widely distributed in the suture, endocranial, and ectocranial regions, with a small number present in mature bone tissue (Figures 3H,H′). At PN14, a small number of tdTomato-positive cells were mainly distributed in the endocranial and ectocranial regions, while the number of tdTomato-positive cells in the suture decreased. Meanwhile, Sp7 and tdTomato double-positive cells were observed at the osteogenic front (Figures 3I,I’). By PN21, tdTomato signal was predominantly present in the bilateral parietal bones, with no tdTomato-positive cells in the suture including the osteogenic front (Figures 3J,J’). Quantitative analysis revealed that from PN9 to PN28, the number of tdTomato-positive cells in the sagittal suture gradually decreased until disappearing, whereas their number in the bilateral parietal bones continued to increase (Figures 3K,L). These results indicate that the proliferating cells within the suture exhibit features of TACs, can contribute to Pre-osteoblasts, Osteoblasts, Endocranial cells, and Ectocranial cells, but lack self-maintenance and renewal capacity.

In addition, we noted that tdTomato-positive cells in the suture contributed to the endocranial and ectocranial cells, indicating that these proliferating cells serve as their progenitors, which is consistent with the CytoTRACE2 analysis based on Smart-seq3 sequencing.

### 3.5 Single-cell sequencing analysis identifies ETS-related gene (*Erg*) as a key transcription factor in SuSCs and delineates its regulated signaling network

**FIGURE 4:**
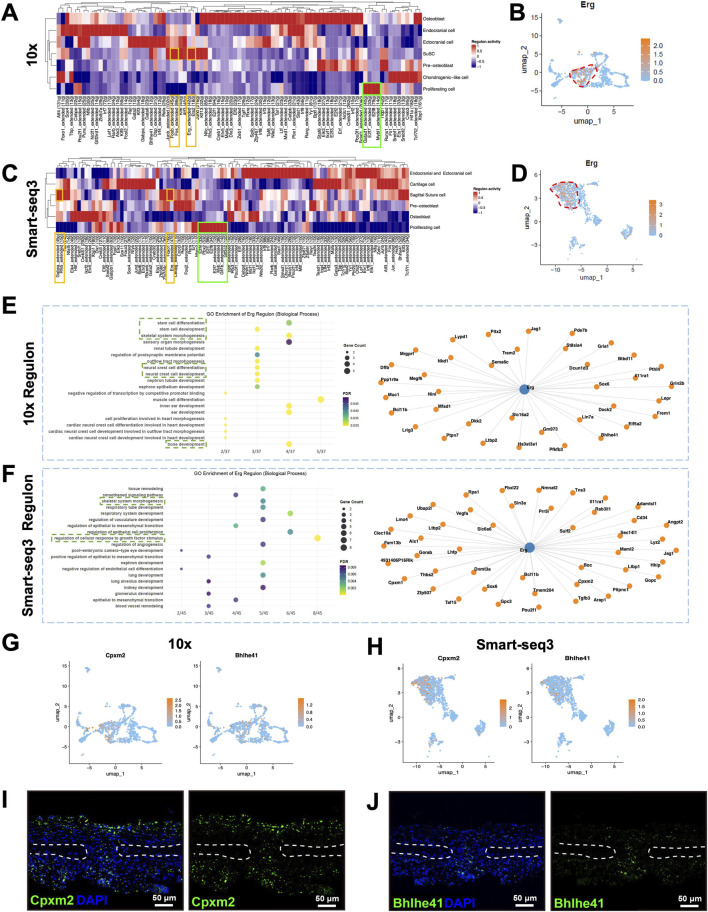
SCENIC analysis within SuSCs in sagittal suture. **(A)** The SCENIC analysis reveals regulon activity in SuSCs based on 10x data. **(B)** The UMAP visualization of *Erg* in osteogenic related cells based on 10x data. **(C)** The SCENIC analysis reveals regulon activity in SuSCs based on Smart-seq3 data. **(D)** The UMAP visualization of *Erg* in osteogenic related cells based on Smart-seq3 data. **(E)** The GO enrichment analysis on *Erg* target genes based on 10x data. **(F)** The GO enrichment analysis on *Erg* target genes based on Smart-seq3 data. **(G)** The UMAP visualization of *Cpxm2* and *Bhlhe41* in osteogenic related cells based on 10x data. **(H)** The UMAP visualization of *Cpxm2* and *Bhlhe41* in osteogenic related cells based on Smart-seq3 data. **(I)** The *in situ* hybridization of *Cpxm2* transcript in sagittal suture of PN7 wild type mice. **(J)** The *in situ* hybridization of *Bhlhe41* transcript in sagittal suture of PN7 wild type mice.

To delineate the key gene regulating SuSCs, we employed the SCENIC package. In the framework of SCENIC, the regulon was identified with one transcription factor with its high-confidence targets genes. And the heatmap of regulon activity represent how strongly each regulon active across all the cell type. In the heatmap of regulon activity, we screened for regulons with elevated activity exclusively in SuSCs. Our initial screening identified *Fosb*, *Fos*, *Erg*, and *Ets2* regulons in the 10x group (Figure 4A, yellow solid boxes), and *Foxo1*, *Erg*, *Gabpa*, and *Rfx5* regulons in the Smart-seq3 group (Figure 4C, yellow solid boxes) as highly active in SuSCs. Subsequent UMAP visualization of transcription factor expression patterns revealed that only *Erg* displayed specific enrichment in SuSC (Figures 4B,D; Supplementary Figure S9). Subsequently, we performed GO enrichment analysis on *Erg* target genes. Based on 10x sequencing data, the MSCs regulation related biological process regulated by *Erg* target genes included stem cell differentiation (*Pitx2*, *Jag1*, *Sema6c*, *Sox6*), neural crest cell development and neural crest cell differentiation (*Pitx2*, *Jag1*, *Sema6c*), and stem cell development (*Pitx2*, *Jag1*, *Sema6c*); and bone development related biological process included bone development (*Pitx2*, *Frem1*, *Lepr*, *Pthlh*) and skeletal system morphogenesis (*Frem1*, *Pthlh*, *Sox6, Jag1*) (Figure 4E). Based on Smart-seq3 sequencing data, the MSCs regulation related biological process regulated by *Erg* target genes included regulation of cellular response to growth factor stimulus (*Gpc3*, *Sulf2*, *Tgfb3*, *Hhip*, *Ltbp1*, *Sin3a*, *Vegfa*), and bone-related biological process included skeletal system morphogenesis (*Tgfb3*, *Hhip*, *Sox6*) (Figure 4F). These results suggest that *Erg* is a transcription factor highly expressed in SuSCs, and its downstream target genes participate in biological process governing MSCs and bone morphogenesis.

Further UMAP analysis of the expression patterns of *Erg* target genes revealed that *Bcl11b*, *Dkk2*, *Ltbp2*, *Bhlhe41*, *Nkd1* and *Jag1* (10x), as well as *Jag1*, *Bcl11b*, *Cpxm2*, and *Hhip* (Smart-seq3), are all preferentially expressed in SuSCs (Figures 4G,H; Supplementary Figures S10, S11). Among these, except for *Cpxm2* and *Bhlhe41*, the remaining genes have been reported to play key roles in craniofacial and suture development; meanwhile, *Ltbp2* knockout mice reportedly show no obvious phenotype (Bodmer et al., 2024). And the function of Naked cuticle (*Nkd*) has been proved that is dispensable for craniofacial develop (Zhang et al., 2007). Furthermore, *in situ* hybridization confirmed the expression patterns of *Cpxm2* and *Bhlhe41* in the sagittal suture: at PN7, both genes are expressed in the central region of mesenchymal, with positive signals detected in partial region of ectocranial and endocranial cells, and no expression at the osteogenic fronts or within the parietal bone (Figures 4I,J).

### 3.6 Single-cell sequencing reveals key transcription factors of proliferating cells and their downstream signaling networks

To analyze key transcription factors in proliferating cells and their regulated signaling networks, we applied the aforementioned approach to preliminarily screen regulons with relatively high activity in Proliferating cell in both the 10x and Smart-seq3 datasets (Figures 4A,C, green solid boxes).

**FIGURE 5:**
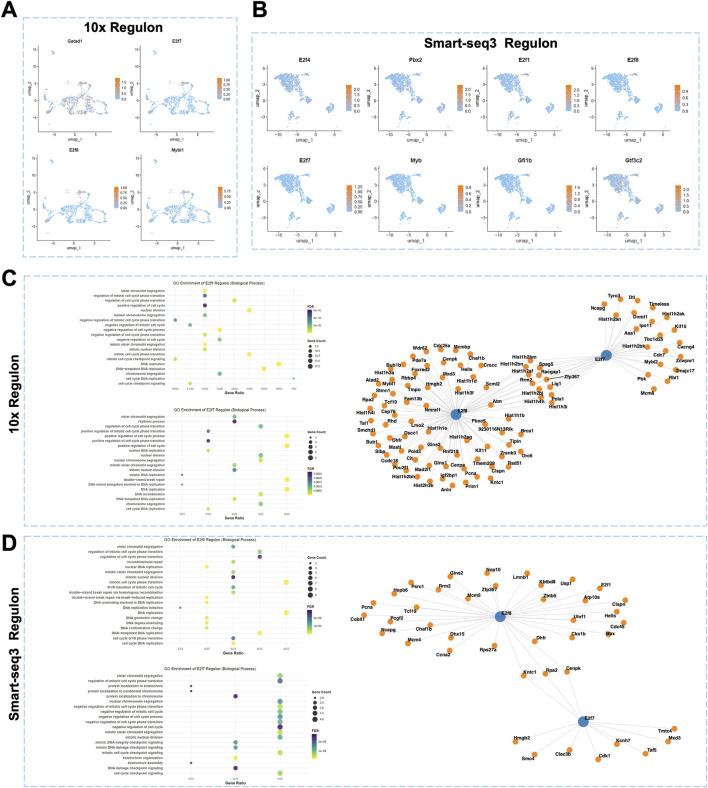
SCENIC analysis within Proliferating cell in sagittal sutures. **(A)** The UMAP visualization of key transcription factors in proliferating cells based on 10x data. **(B)** The UMAP visualization of key transcription factors in proliferating cells based on Smart-seq3 data. **(C)** The GO enrichment analysis on *E2f8* and *E2f7* downstream target genes based on 10x data. **(D)** The GO enrichment analysis on *E2f8 and E2f7* downstream target genes based on Smart-seq3 data.

Further UMAP analysis showed that, in the 10x dataset, *E2f7*, *E2f8*, and *Mybl1* expression is largely restricted to Proliferating cell (Figure 5A). However, in the Smart-seq3 dataset, *E2f7* and *E2f8* exhibit relatively low expression in Proliferating cell (Figure 5B). We then performed GO enrichment analyses on the downstream target genes of *E2f7* and *E2f8*. The results indicated that their target genes are highly enriched in biological process closely related to the cell cycle and DNA replication (Figures 5C,D). GO analysis of *Mybl1* downstream genes only revealed two enriched biological processes: regulation of dephosphorylation and regulation of protein dephosphorylation involving *Cdca2* and *Mastl*; these are not shown. Collectively, these findings indicate that *E2f7* and *E2f8* are regulons specifically expressed in Proliferating cells within the sagittal suture mesenchyme, and their downstream signaling networks are involved in cell proliferation control, suggesting that these two factors may be key regulators of proliferating cell amplification.

## 4 Discussion

In this study, we initially followed published approaches to profile sagittal suture directly with 10x Genomics, and identified osteogenesis-related cells using their marker genes (Farmer et al., 2021; Holmes et al., 2021; Li et al., 2023b). Although this approach resulted in suboptimal capture of osteogenic cell populations, it provided a comprehensive range of cell populations among the sample, which was consistent with prior reports (Li et al., 2023b). Given that *Gli1*-positive cells in craniofacial sutures are the principal MSCs contributing to craniofacial bone formation (Zhao et al., 2015), we performed FACS-based Smart-seq3 of *Gli1*-positive cells and their progeny from PN7 sagittal sutures. Overall, the mean nFeature RNA and mean nCount RNA of the Smart-seq3 group were superior to those of the 10x group, indicating that Smart-seq3 had higher sequencing depth. In terms of the number of captured cells, the total number of cells captured by the 10x group was significantly greater than that by the Smart-seq3 group. Although the absolute number of osteogenic related cells captured by the two groups was similar in this study, the Smart-seq3 group achieved higher capture efficiency for osteogenic related cells than the 10x group, and the number of target cells could be further efficiently increased by collecting more samples. In addition, the 10x group captured a larger number of non-osteogenic cells, which was helpful to comprehensively understand the gene expression profiles of various cell populations in the sagittal suture.

CytoTRACE2 analysis based on 10x dataset suggested that endocranial and ectocranial cells were at relatively low differentiation states, which contradicted predictions from the Smart-seq3 dataset. Using lineage tracing in *Ki67-Cre* ^*ERT2*^ *;tdTomato* mice, we confirmed that a subset of proliferating cells directly differentiated into both endocranial and ectocranial populations, indicating that proliferating cells were in fact less differentiated than these two lineages. We infer that the discrepancy between the two platforms likely stems from differences in sequencing depth. Therefore, even though both sequencing methods yield cellular clustering results consistent with prior findings (Farmer et al., 2021; Holmes et al., 2021), the superior sequencing depth and better targets cells capture efficiency of Smart-seq3 provides a crucial foundation for subsequent analyses, like Cytotrace2, following cell clustering. This suggests that Smart-seq3 can specifically conduct in-depth analysis of the gene biological profiles of rare cell populations, providing technical support for the refined analysis of cell types and subpopulations (Hagemann-Jensen et al., 2020). However, it only performs in-depth analysis of target cells, which might hinder the understanding of interactions between all cell populations within the microenvironment. In contrast, the 10x process large-scale cell samples simply and efficiently (Ziegenhain et al., 2017; Wang et al., 2021), enabling high-throughput cell capture and identification (Ziegenhain et al., 2017; Ashton et al., 2021). Thus, the two sequencing technologies are complementary in their application scenarios, and the selection should be based on the research objectives.

In previous studies, Zhao et al. demonstrated that MSCs in the apical region of mouse incisors undergo rapid population expansion via a transitional state termed TACs, thereby providing a robust cellular foundation for the lifelong regeneration of tissues such as dental pulp and dentin (Li et al., 2015; Zhao et al., 2018). In the present study, we first detected the population of cells with high proliferative activity using the EdU assay. In the early stage after EdU administration, a certain proportion of EdU-positive cells were detected in the lateral region of the sagittal suture; however, the proportion of EdU-positive cells decreased significantly at 8 h and 12 h. These results indicated that this cell population exhibited rapid proliferation and a transient existence, which was an essential characteristic of TACs identity. Owing to the rapid metabolic rate of EdU *in vivo*, long-term tracing could not be achieved. Meanwhile, we observed that at 2 h and 4 h post-EdU labeling, approximately 60% of the EdU-labeled cell population were co-labeled with Ki67. This suggested that Ki67 could also mark this cell population with high proliferative potential during suture development, which was consistent with the results of *Mki67 in situ* hybridization in the mesenchyme of the sagittal suture lateral region. Further trajectory analysis of the Ki67-positive cells revealed that their progeny was widely distributed across tissues outside the central region of suture mesenchyme, indicative of their capacity to differentiate into osteoblasts, endocranial cells, and ectocranial cells. Collectively, these findings indicate that this population fulfills the core criteria of TACs: localization adjacent to SuSCs, a differentiation state second only to SuSCs, rapid proliferation kinetics, and a transient cellular fate. Thus, our study provides the first identification of TACs within cranial suture mesenchyme, revealing a “quiescence-proliferation-differentiation” hierarchical regulatory paradigm wherein SuSCs orchestrate suture formation via TACs.

The Wnt signaling pathway plays a pivotal role in the development of craniofacial sutures. Abnormal hyperactivation of the Wnt/β-catenin pathway drives aberrant osteogenic lineage commitment of MSCs and excessive proliferation of pre-osteoblasts, leading to craniosynostosis (Yu et al., 2005; Mirando et al., 2010). *Sfrp2*, a key modulator of Wnt signaling, inhibits pathway activation by competitively binding Wnt ligands and disrupting their interaction with Frizzled receptors (Bovolenta et al., 2008; Mii and Taira, 2011). In addition, *Mfap4* encodes an extracellular matrix glycoprotein involved in microfibril assembly, elastogenesis, and tissue homeostasis. While dysregulated *Mfap4* expression is primarily associated with fibrosis in parenchymal organs (Holm et al., 2015; Zhou et al., 2020), its role in bone or MSCs remains poorly characterized. In this study, at PN7 and PN28, *Sfrp2* and *Mfap4* transcripts were highly in SuSCs but absent in pre-osteoblasts or osteoblasts, suggesting they were not involved in osteogenic differentiation. Notably, their expression was spatially restricted not only to the suture center but also extended bilaterally into non-fused regions like endocranial and ectocranial areas. We hypothesize that *Sfrp2* and *Mfap4* may critically regulate SuSCs homeostasis or stemness maintenance. However, their functional significance in ectocranial and endocranial cells warrants further investigation.

*Erg* is a member of the ETS family of transcription factors, plays a pivotal role in regulating diverse critical biological processes, and has been identified as a marker gene for progenitor cell populations in embryonic sutures (Farmer et al., 2021). In this study, we observed that the expression of *Erg* exhibits high specificity in SuSCs. Enrichment analysis of *Erg* downstream target genes revealed their predominant association with MSCs differentiation, bone development, and other biological processes closely linked to craniofacial morphogenesis. Notably, several genes, including *Hhip* (Holmes et al., 2021), *Gpc3* (Anna K et al., 2007; Dwivedi et al., 2013; Bariana et al., 2018), *Tgfb3* (Opperman et al., 2002; Lyn Chong et al., 2003; Yen et al., 2010), and *Jag1*(Alagille et al., 1987), have been implicated in cranial suture development. Furthermore, UMAP and *in situ* hybridization analysis identified carboxypeptidase X, M14 family member 2 (*Cpxm2*) and basic helix-loop-helix family, member e41 (*Bhlhe41*) as exhibiting specific expression in SuSCs, suggesting their potential key roles in suture development and SuSCs regulation. Future investigations into *Erg* and its downstream targets, *Cpxm2* and *Bhlhe41*, may provide novel insights into the molecular mechanisms and signaling networks underlying cranial suture formation and maintenance.

The E2F family of transcription factors plays a pivotal role in regulating diverse biological processes, including cell proliferation, differentiation, cell cycle progression, and apoptosis (Lammens et al., 2009). *E2f7* and *E2f8* are structurally similar, and function synergistically in cell cycle regulation (Logan et al., 2005). During embryonic development, double knockout of *E2f7* and *E2f8* triggers severe abnormalities such as widespread apoptosis, vascular dilation, and hemorrhage, culminating in embryonic lethality by E11.5 in mice (Li et al., 2008). Moreover, as downstream effectors of the Wnt/β-catenin signaling pathway, *E2f7* and *E2f8* also govern hepatocyte mitosis and proliferation (Jin et al., 2022). In this study, *E2f7/8* regulon exhibited highly active in TACs, with their downstream gene networks enriched in critical processes such as apoptosis, mitosis, and DNA replication. Additionally, we uncovered a unique expression pattern of *E2f7/8* in TACs population. These results position *E2f7/8* as a promising target for elucidating the mechanisms underlying the rapid expansion of TACs within the suture stem cell niche.

In summary, our study systematically dissects the cellular heterogeneity of sagittal suture progenitors and identifies key regulatory genes regulating SuSCs and TACs, which is essential for suture formation and homeostatic maintenance. In the context of regenerative medicine, understanding the biological properties of SuSCs and TACs reveals their translational potential as cell candidates for cranial bone defect therapy, because the tissue-resident SuSCs and TACs may enable spatially controlled bone regeneration, thus avoiding unintended suture fusion or bone overgrowth. Together, our work bridges the gap between suture developmental biology and clinical applications, providing a translational framework for both craniosynostosis treatment and cranial bone regeneration.

## Data Availability

The datasets generated for this study can be found in the NCBI Gene Expression Omnibus under the accession number GSE309200 (10x), and China National Center for Bioinformation under the accession number CRA033409 (Smart-seq3).
