# Supplementary material for: Single-cell RNA sequencing revealed cell heterogeneity in sagittal suture mesenchyme
Source: Front Cell Dev Biol. 2026 Jan 20;14:1725375. doi: 10.3389/fcell.2026.1725375 (PMC12864488; doi:10.3389/fcell.2026.1725375)
Supplement: Supplementary file 1 [file DataSheet1.docx]

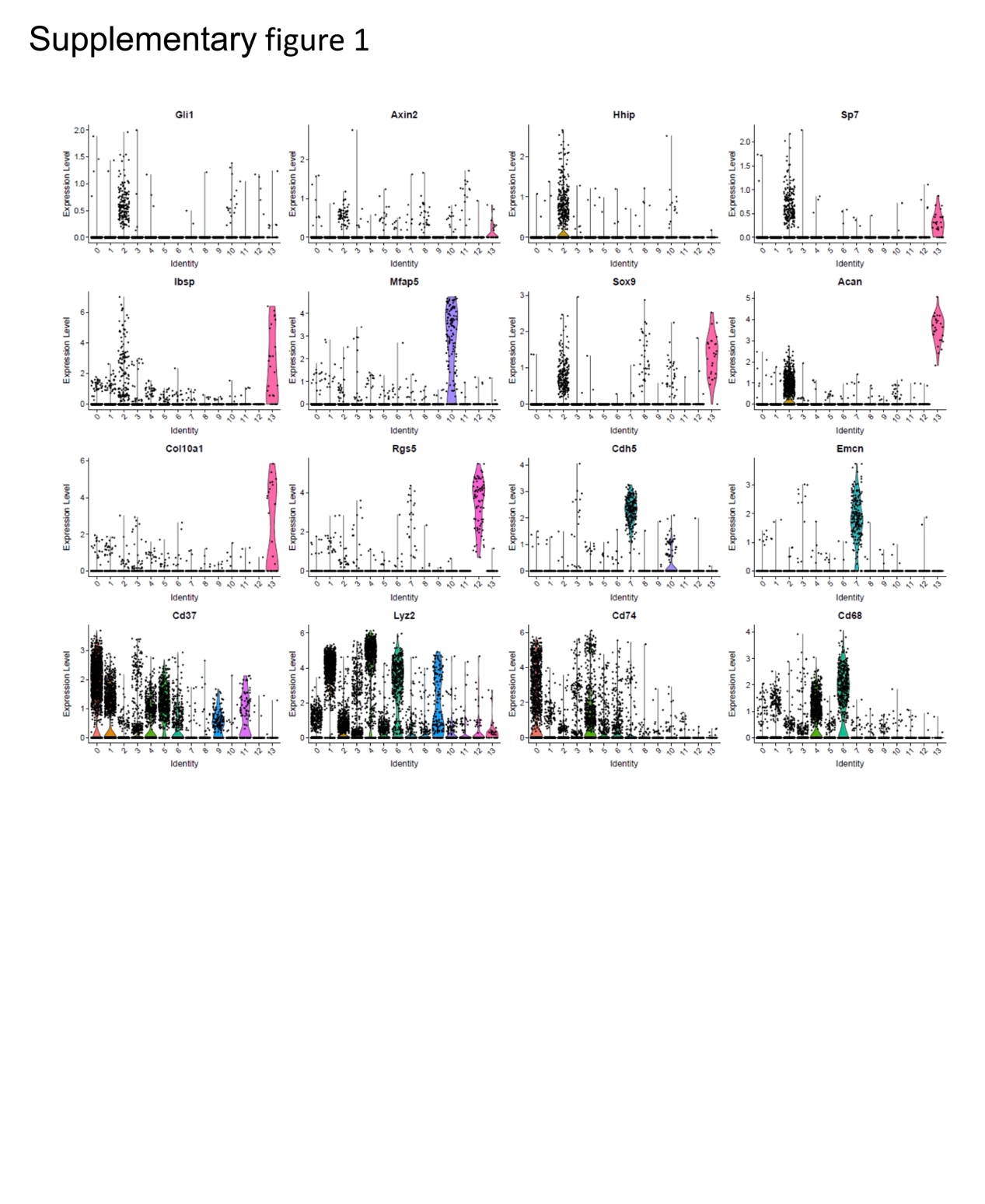
Supplementary Material

**Supplementary Figure 1.** Signature genes in the sagittal suture cell population of 10x group.


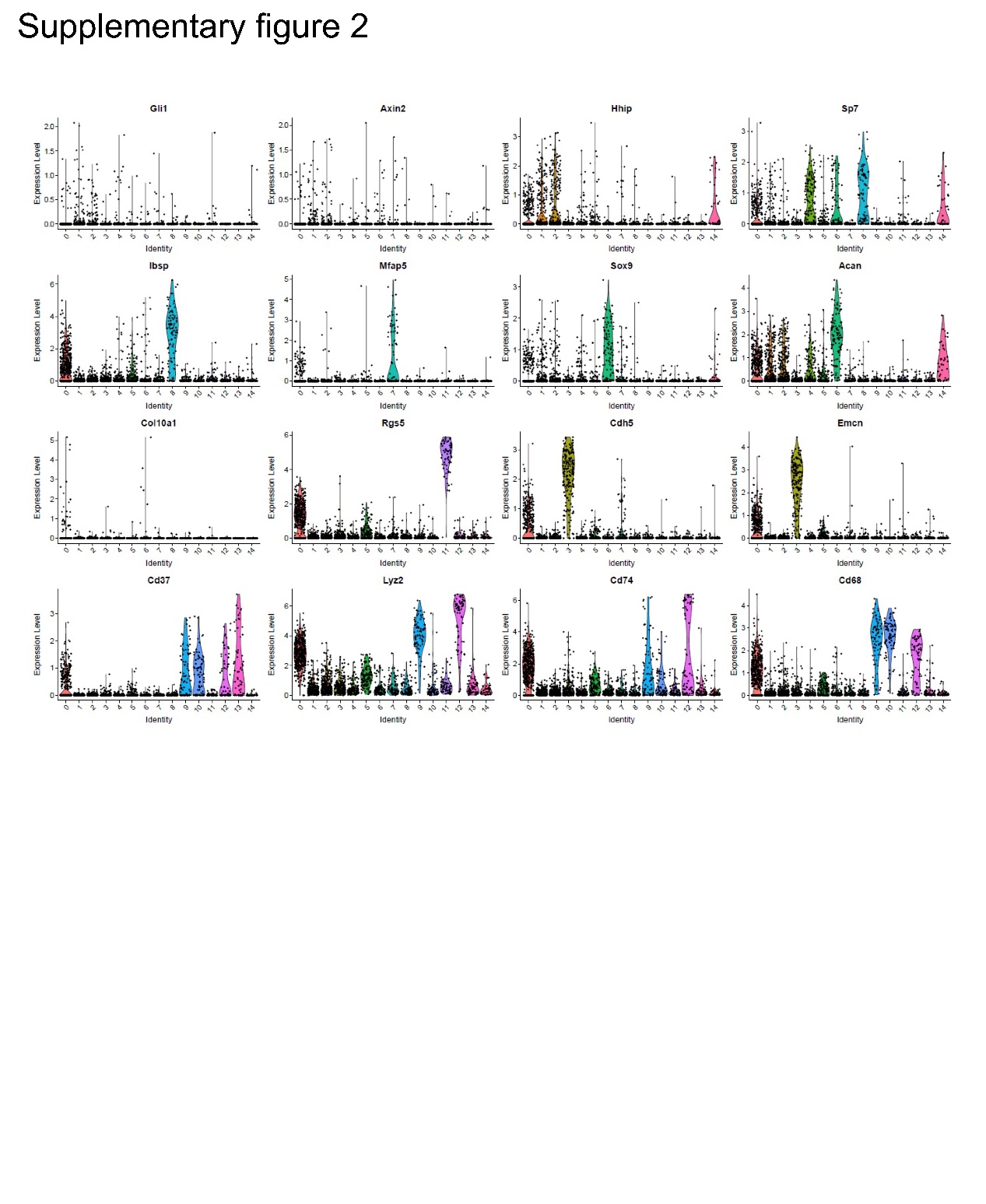


**Supplementary Figure 2.** Signature genes in the sagittal suture cell population of Smart-seq3.


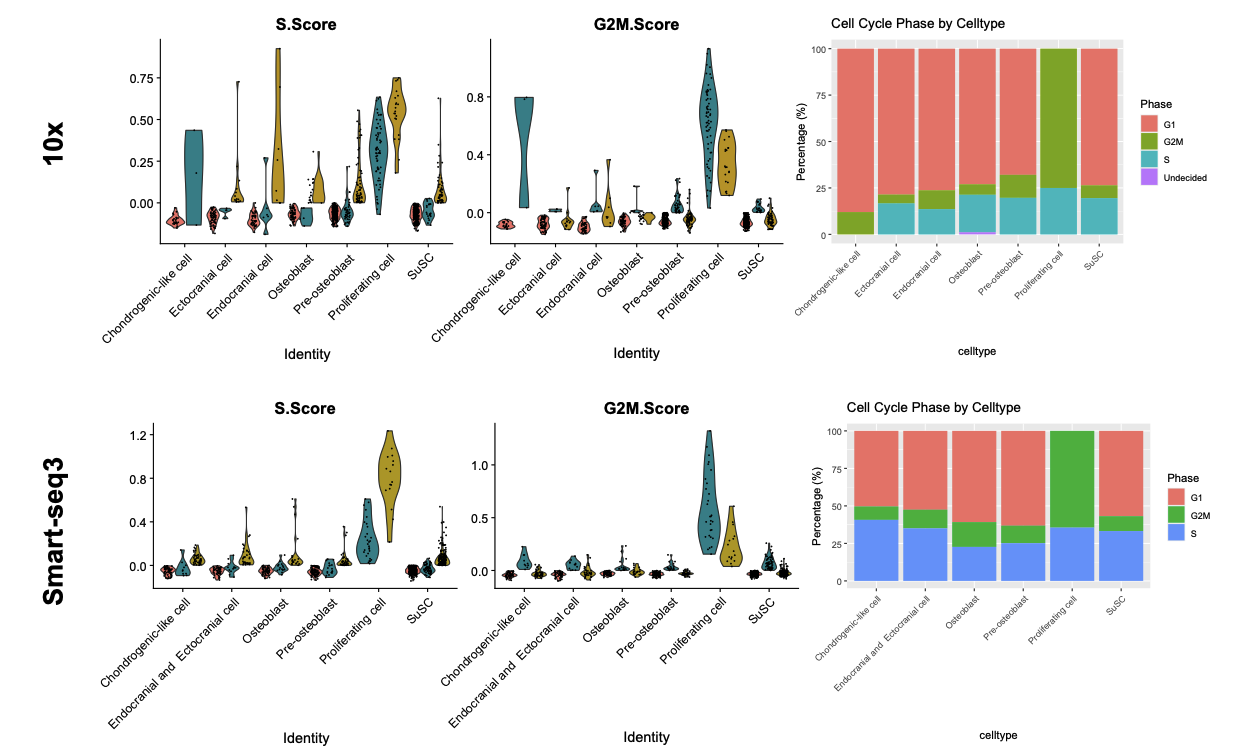
**Supplementary Figure 3.** Cell cycling scoring analyses based on 10x and Smart-seq3.


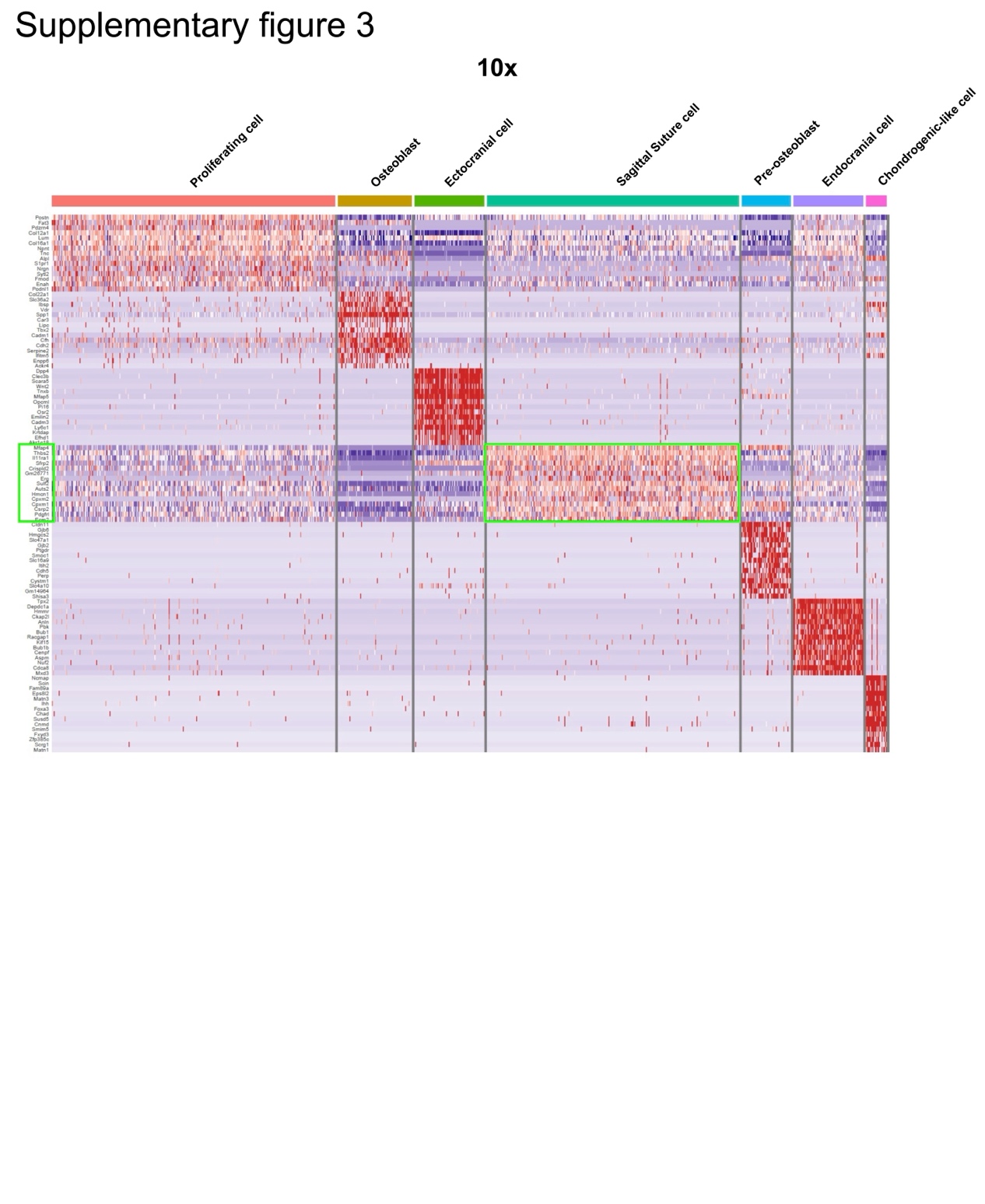


**Supplementary Figure 4.** Top 15 genes of each subcluster of osteogenic-related cells in 10x group.


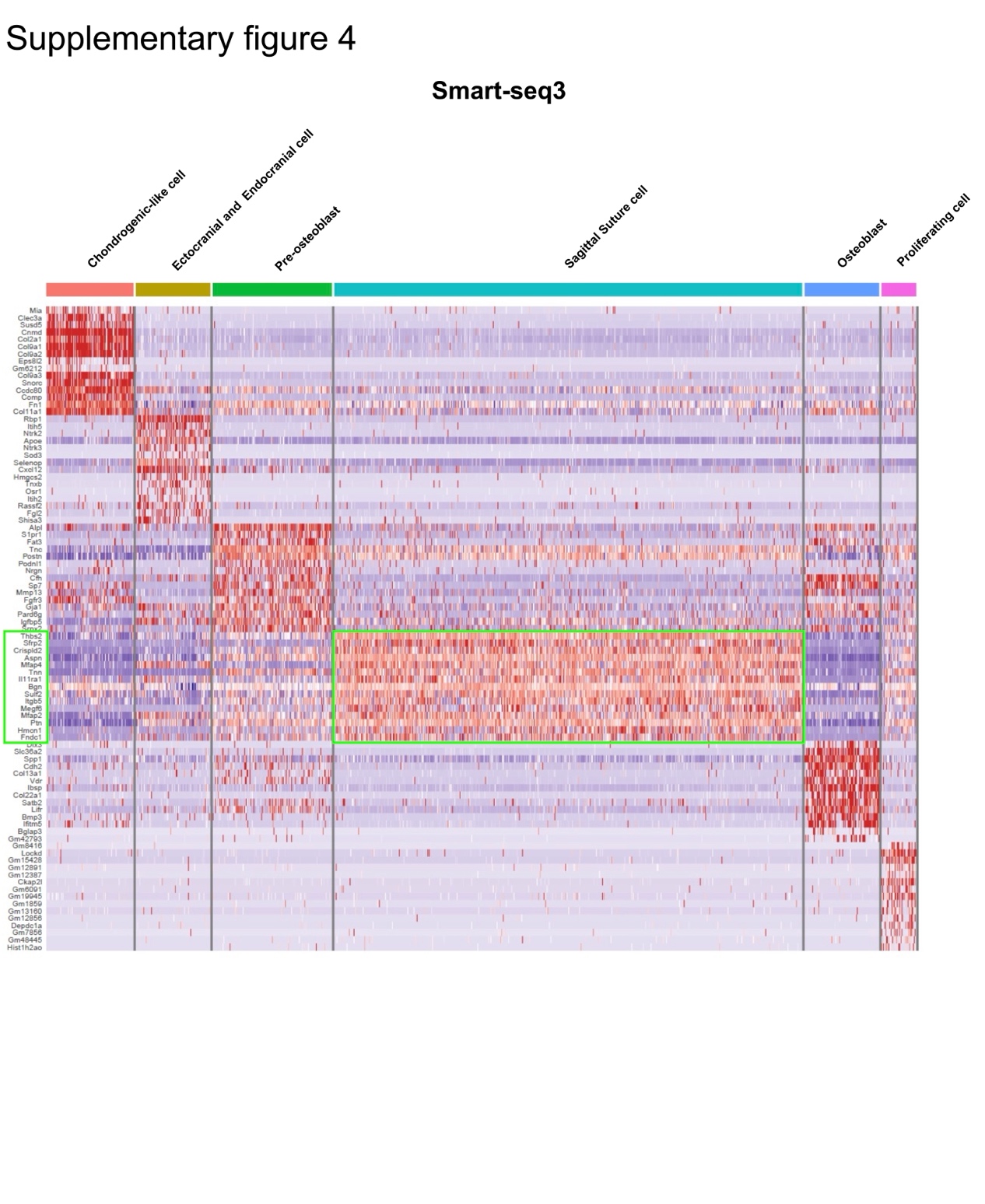


**Supplementary Figure 5.** Top 15 genes of each subcluster of osteogenic-related cells in Smart-seq3 group.


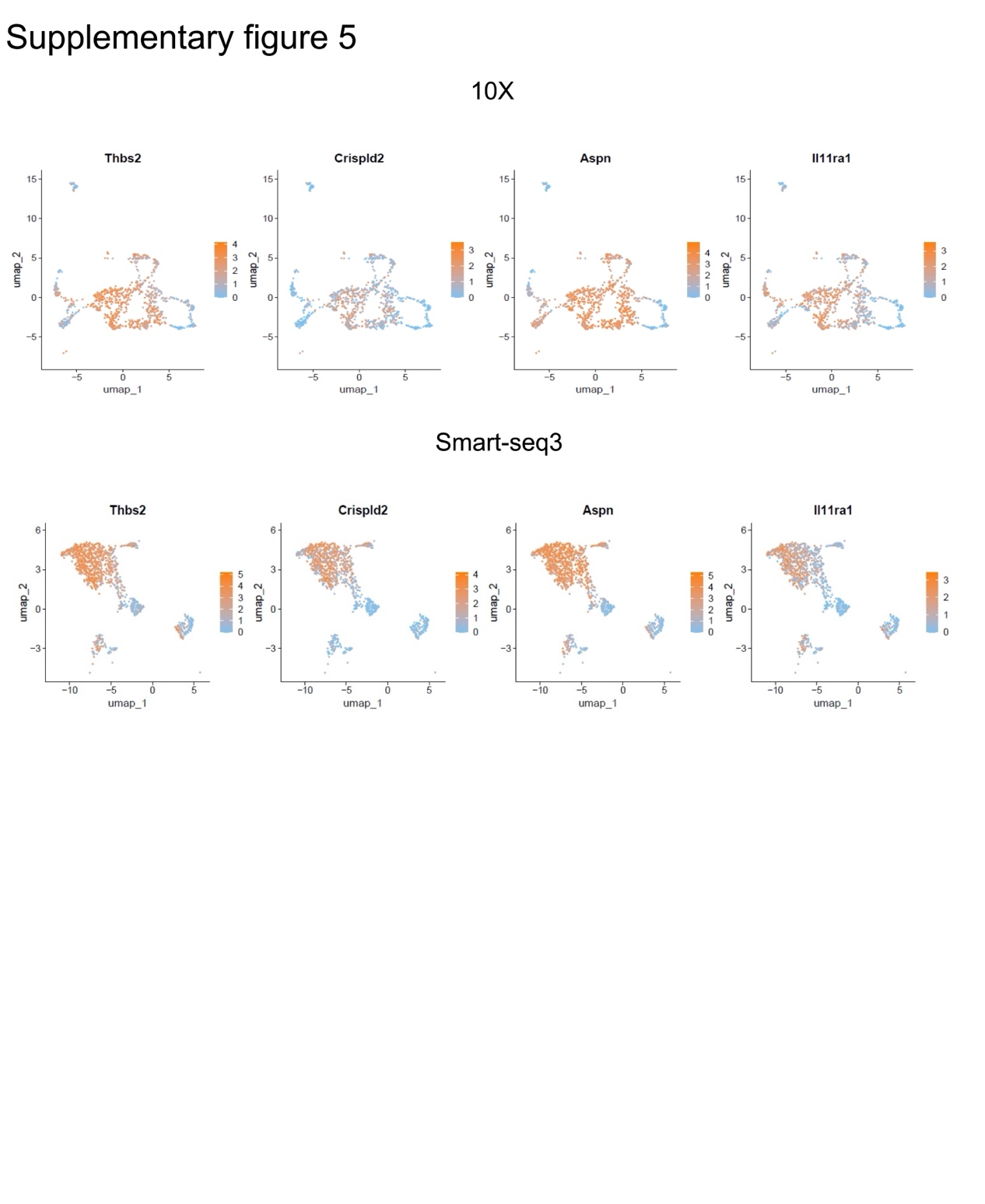


**Supplementary Figure 6.** Feature plot of another top genes of both 10x and Smart-seq3 group, respectively.


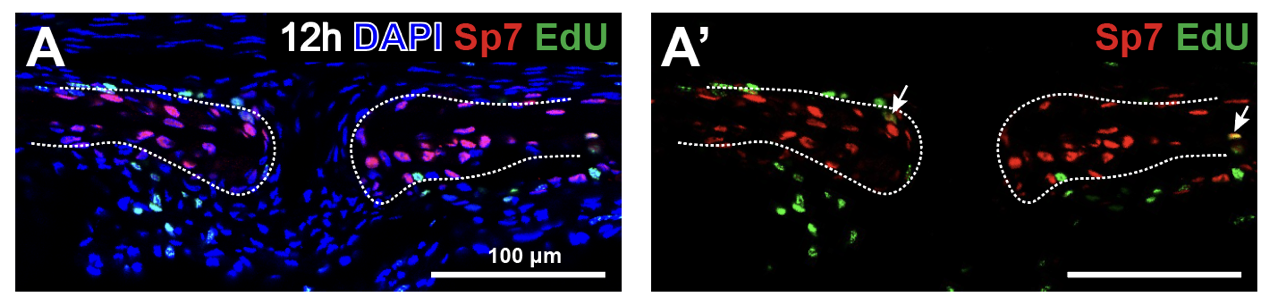
**Supplementary Figure 7.** The results of co-labeling of EdU and Sp7 in the sagittal suture at 12 h after intraperitoneal injection of EdU. Arrows indicate EdU and Sp7 double-positive cells within parietal bones.

**
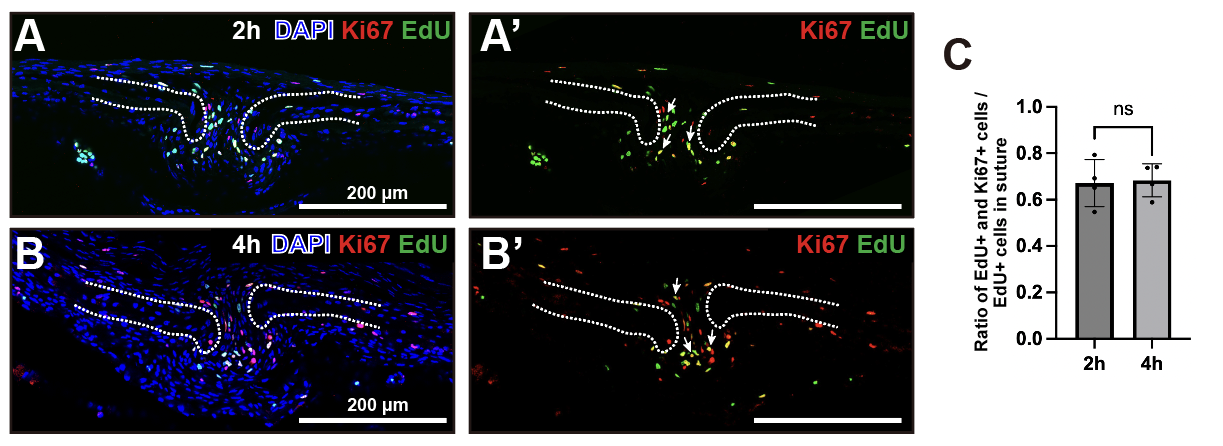
Supplementary Figure 8.** The results of co-labeling of EdU and Ki67 in the sagittal suture and the statistical results at 2 h and 4 h after intraperitoneal injection of EdU. Arrows indicate EdU and Ki67 double-positive cells within sagittal suture.


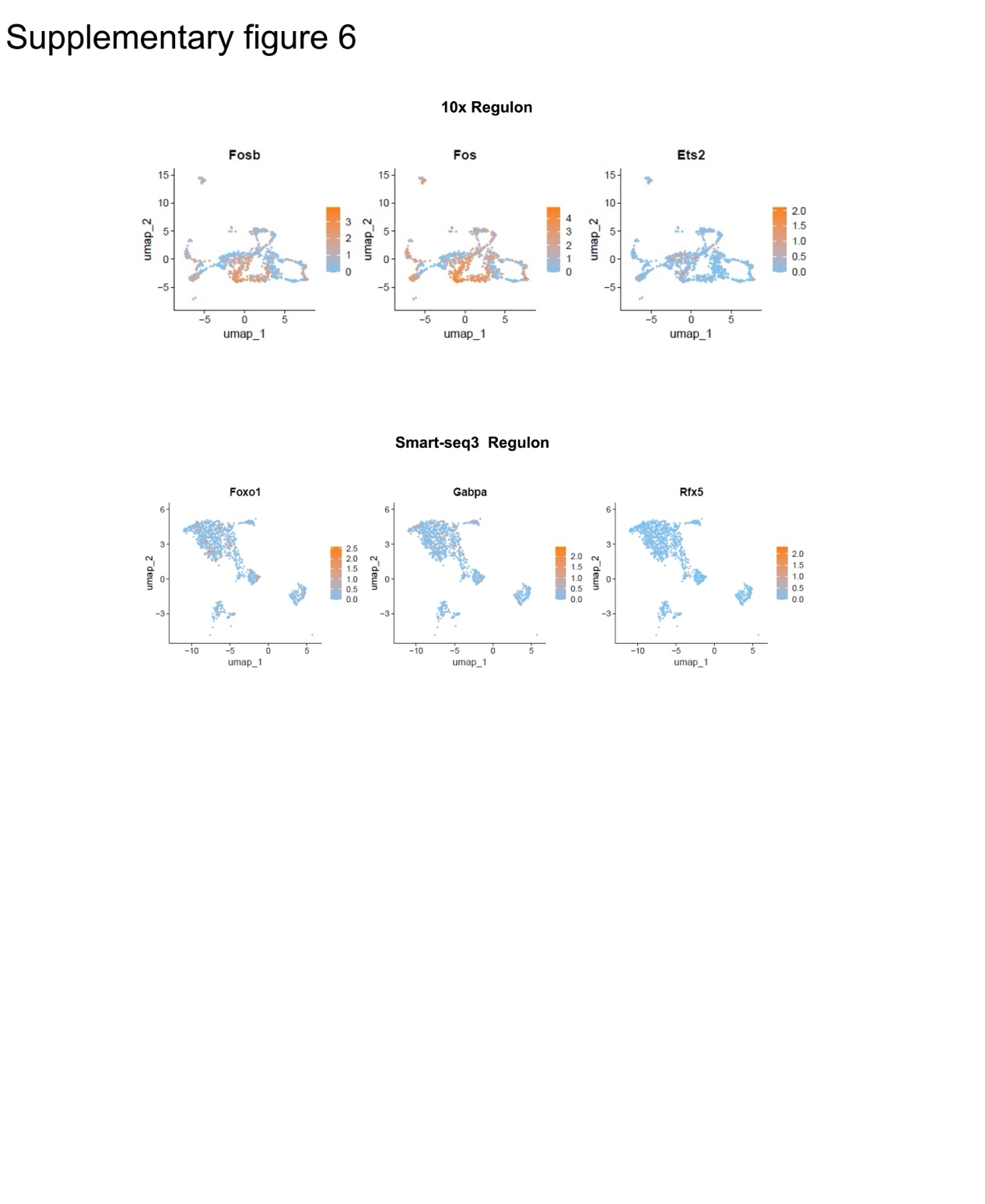


**Supplementary Figure 9.** Feature plots of transcription factors whose regulons are highly active in the SuSCs.

**
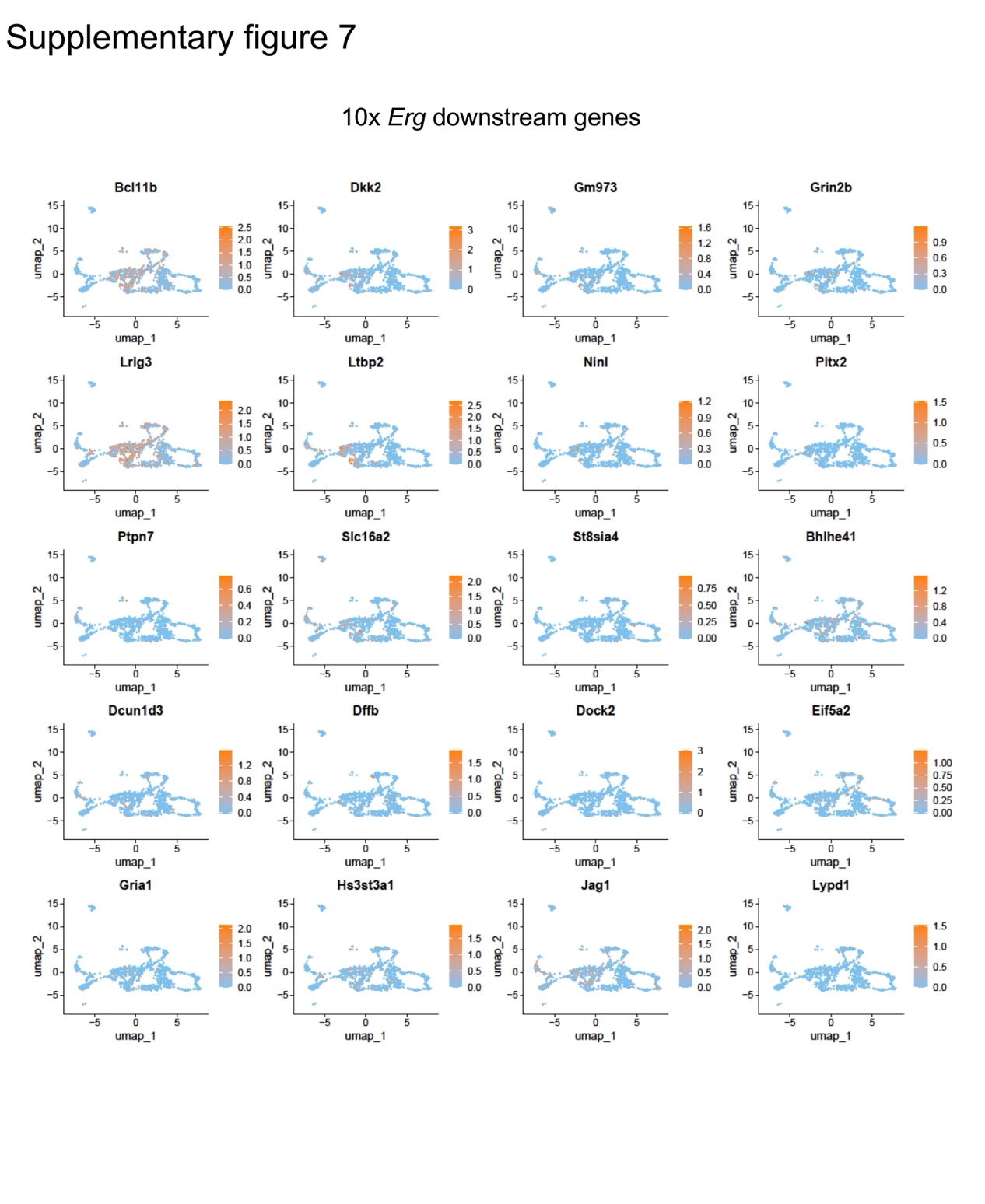
**

**
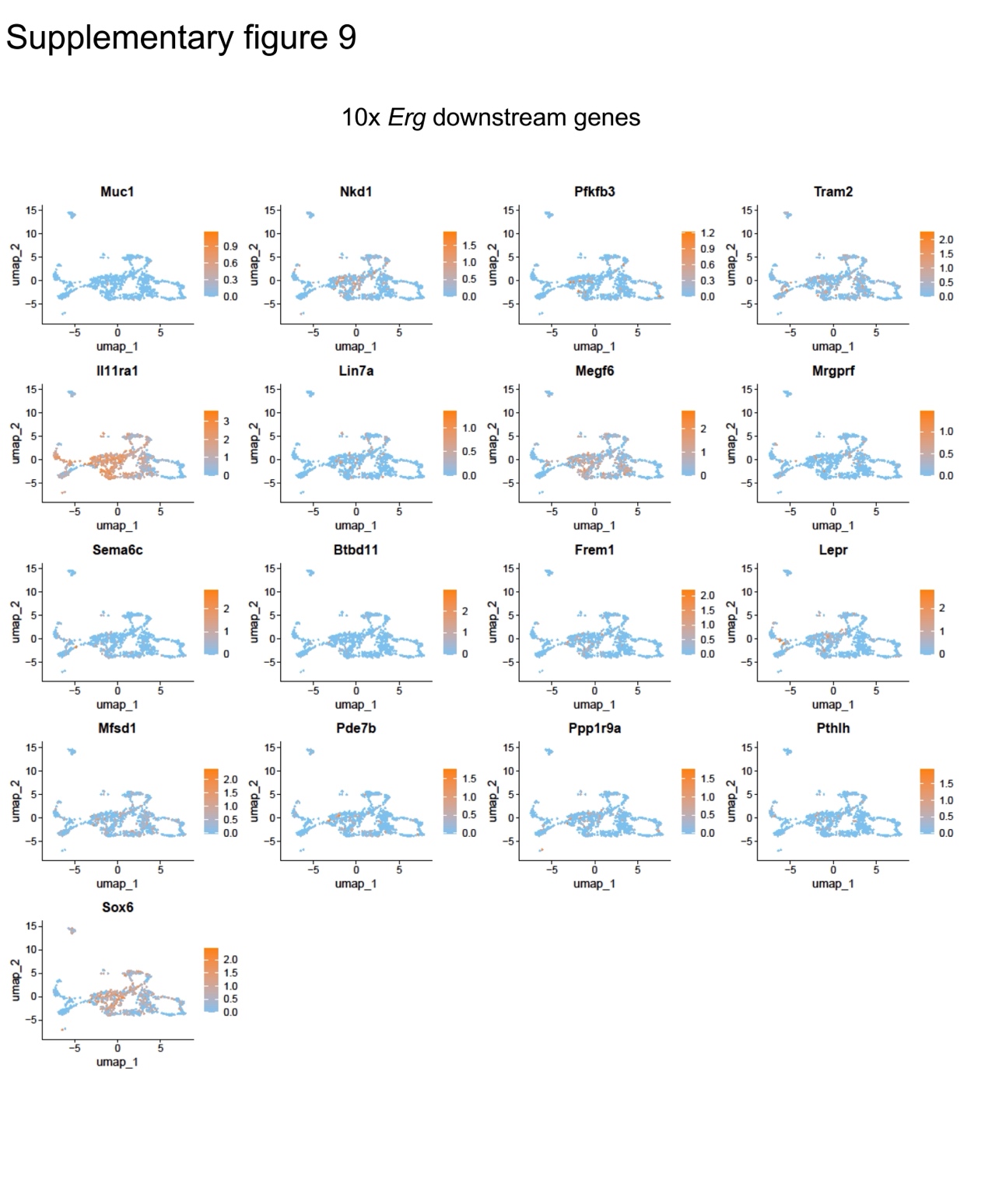
Supplementary Figure 10.** Feature plots of all *Erg* target genes in 10x group.


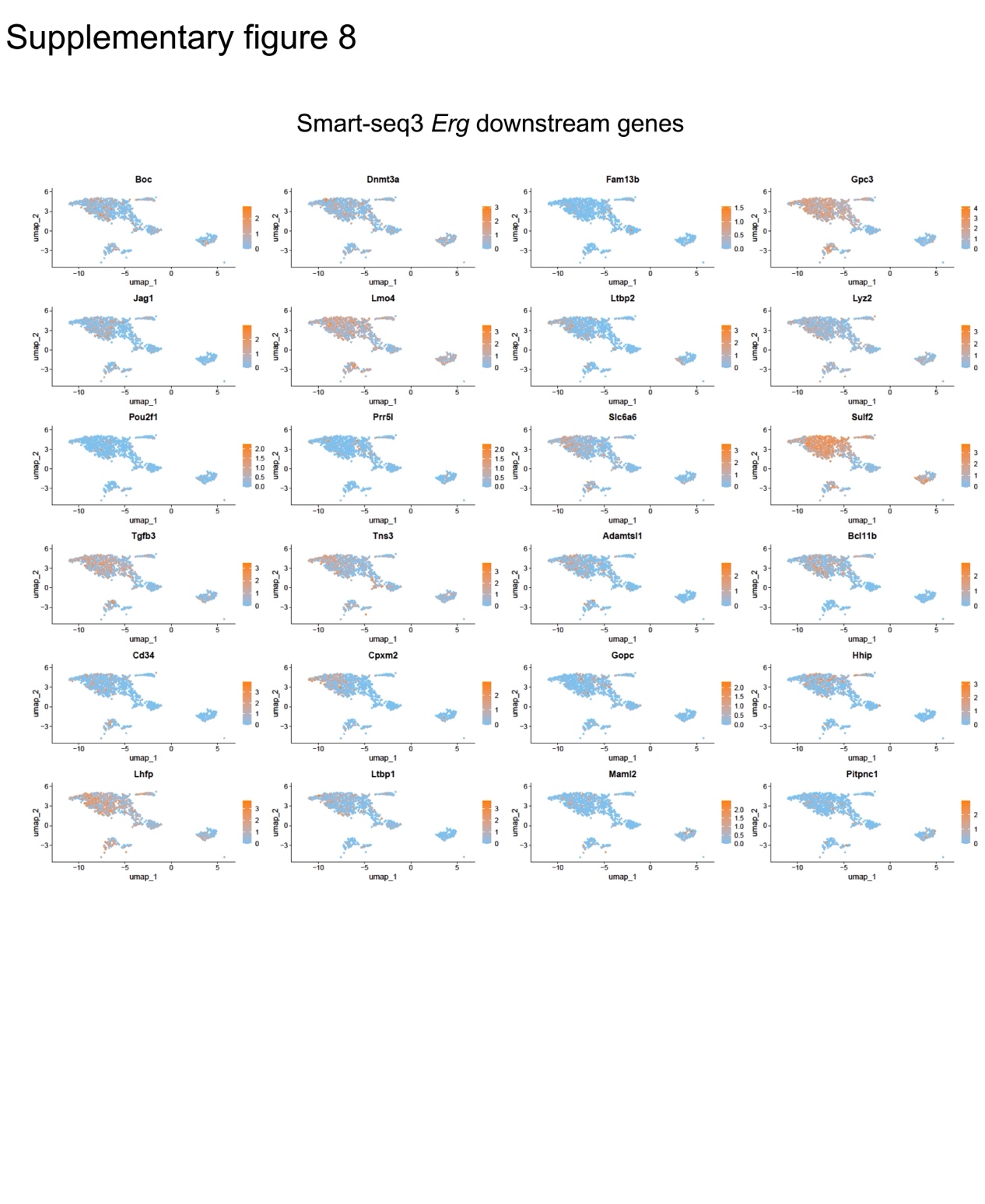


**
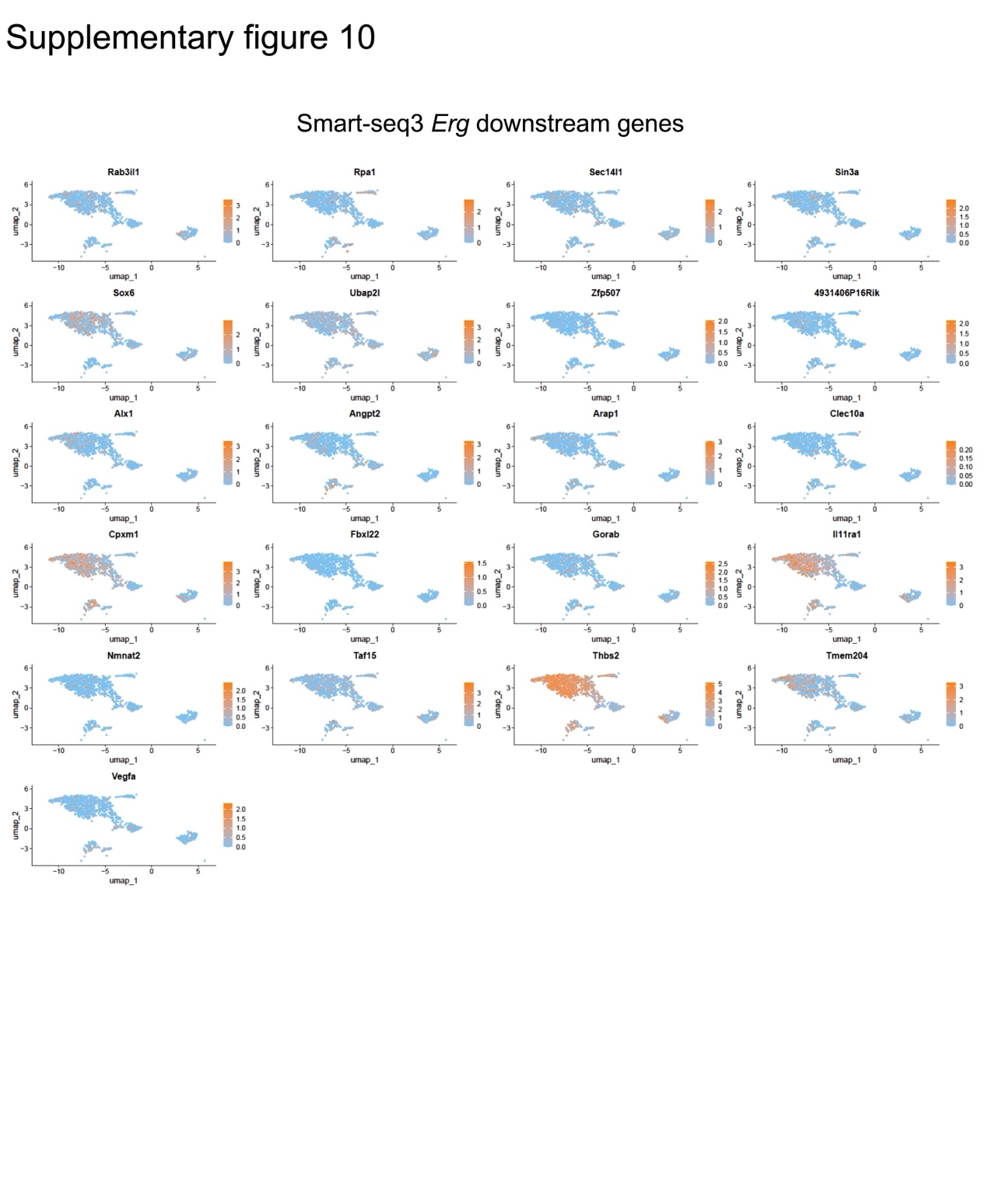
Supplementary Figure 11.** Feature plots of all *Erg* target genes in Smart-seq3 group.

**
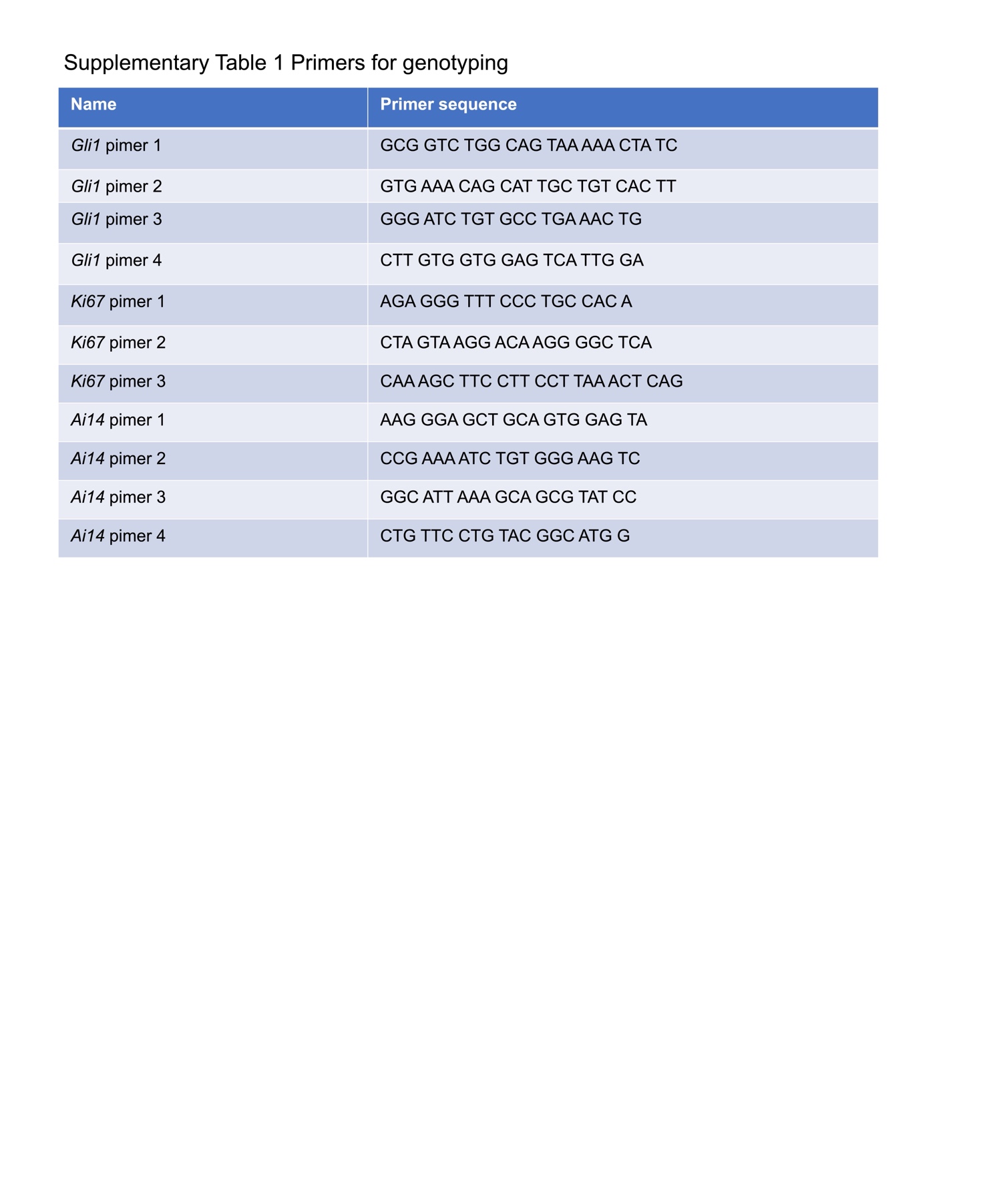
Supplementary Table 1.** Primers for genotyping
